# Effectiveness of an Integrated Community-Based Livelihood and Rehabilitation Intervention on the Social Capital of Caregivers of Children with Cerebral Palsy: Secondary Analysis of an Existing Cluster Randomized Controlled Trial in Rural Bangladesh

**DOI:** 10.3390/children12121687

**Published:** 2025-12-11

**Authors:** Manik Chandra Das, Israt Jahan, Mahmudul Hassan Al Imam, Delwar Akbar, Shafiul Islam, Nuruzzaman Khan, Mohammad Muhit, Nadia Badawi, Gulam Khandaker

**Affiliations:** 1 School of Health, Medical and Applied Sciences, Central Queensland University, Rockhampton, QLD 4701, Australia; manikchandra.das@cqumail.com (M.C.D.); i.jahan@cqu.edu.au (I.J.); gulam.khandaker@health.qld.gov.au (G.K.); 2 CSF Global, Dhaka 1212, Bangladesh; shafinitor09@gmail.com (S.I.); mmuhit@hotmail.com (M.M.); 3 Asian Institute of Disability and Development (AIDD), University of South Asia, Dhaka 1212, Bangladesh; 4 Central Queensland Public Health Unit, Central Queensland Hospital and Health Service, Rockhampton, QLD 4700, Australia; 5 School of Business and Law, Central Queensland University, Rockhampton, QLD 4701, Australia; d.akbar@cqu.edu.au; 6 Department of Population Science, Jatiya Kabi Kazi Nazrul Islam University, Mymensingh 2220, Bangladesh; nuruzzaman.khan@unimelb.edu.au; 7 Nossal Institute for Global Health, Melbourne School of Population and Global Health, The University of Melbourne, Parkville, VIC 3010, Australia; 8 Cerebral Palsy Alliance, Sydney Medical School, The University of Sydney, Camperdown, NSW 2086, Australia; nadia.badawi@health.nsw.gov.au; 9 Grace Centre for Newborn Intensive Care, Sydney Children’s Hospital Network, Westmead, NSW 2145, Australia; 10 Discipline of Child and Adolescent Health, Sydney Medical School, The University of Sydney, Sydney, NSW 2006, Australia

**Keywords:** caregiver, cerebral palsy, children, social capital, lower- and middle-income country, Bangladesh

## Abstract

**Highlights:**

**What are the main findings?**

- Integrated microfinance and community-based rehabilitation (IMCBR) significantly improved caregivers’ overall and structural social capital compared with community-based rehabilitation or standard care.
- Caregivers of children with more severe motor impairments showed smaller gains in social capital.

**What are the implications of the main findings?**

- IMCBR can strengthen caregivers’ social networks, participation, and community engagement in low-resource settings.
- Programs should include targeted support for the caregivers of children with severe CP to ensure equitable benefits and reduce social disadvantages.

**Abstract:**

Background/Objectives: Social capital is a multifaceted concept that comprises structural and cognitive portions, and from the perspective of caregivers, it enables access to assistance and participation, improving well-being in resource-constrained settings. In low- and middle-income countries (LMICs) like Bangladesh, mothers are often the sole carers of children with cerebral palsy (CP), which may affect their social capital and livelihood; however, evidence in this regard is limited. This study assessed the effectiveness of integrated microfinance and community-based rehabilitation (IMCBR) on caregivers’ social capital in rural Bangladesh. Methods: This study was part of a randomized controlled trial (RCT) conducted in Shahjadpur, Sirajganj, with three study arms. Children aged ≤5 years with CP and their primary caregivers were enrolled. Twenty-four clusters (10–14 child–caregiver pairs per cluster) were randomly assigned to Arm-A: IMCBR, Arm-B: community-based rehabilitation (CBR) only, and Arm-C: standard care. Data were collected at the baseline, midline (6 months), and endline (12 months) using a structured questionnaire. Social capital was measured using the Short Adapted Social Capital Assessment Tool (SASCAT), which assesses structural and cognitive dimensions; higher scores indicated greater social capital. The SASCAT was culturally adapted and validated for use in Bangladesh. Descriptive, bivariate, and multivariate analyses were performed. Results: There were 251 dyads enrolled into the trial. At baseline, Arm-A had the lowest social capital scores but showed the greatest improvement by endline (60.0%), followed by Arm-B (54.1%) and Arm-C (6.0%). Structural social capital increased significantly in Arm-A compared with Arm-C (mean difference 2.88; 95% CI: 2.45–3.31; *p* < 0.001) and in Arm-B compared with Arm-C (mean difference 2.46; 95% CI: 2.04–2.87; *p* < 0.001). Cognitive social capital increased the most in Arm-B (10.7%), though group differences were not significant (*p* > 0.05). In Arm-A, improvements in social capital were inversely associated with the child’s Gross Motor Function Classification System (GMFCS) level (β = −0.69; 95% CI: −1.28 to −0.10; *p* < 0.05). Conclusions: IMCBR significantly improved caregivers’ social capital, particularly its structural components, in rural Bangladesh.

## 1. Introduction

Social capital is a complex and multifaceted term. It refers to the network of social relationships, shared norms, trust, and mutual understanding that enables individuals and communities to access support and work collectively toward shared goals [1,2]. Strong social capital is consistently linked to better mental and physical health, greater resilience, and improved quality of life, as it enhances social support, reduces stress, and facilitates access to resources and services [3,4,5,6,7].

For the caregivers of children who require long-term care, such as those with cerebral palsy (CP), maintaining social capital can be particularly challenging due to the high demands of caregiving [3,8,9]. However, it plays an important protective role in buffering psychological distress and supporting overall well-being. For example, a study from Saudi Arabia reported that lower perceived social support was associated with higher caregiver burden, which in turn was significantly correlated with increased perceived stress [9].

CP is a group of neurodevelopmental disorders affecting the motor function, posture, and movement of an individual resulting from brain injury during early development or infancy [10]. It is the leading cause of childhood disability globally, with higher prevalence in low- and middle-income countries (LMICs) compared with high-income countries (HICs) [11,12]. Children with CP often require multidisciplinary care and support for daily functioning; however, in most LMICs, rehabilitation and medical services remain scarce or inaccessible [13]. Data from the Bangladesh CP Register (BCPR) indicate that most children are diagnosed after five years of age and nearly half have never received any rehabilitation services [12,13]. This limited service availability places a substantial burden on primary caregivers—predominantly mothers—restricting their economic participation, affecting their mental well-being and quality of life, and reducing opportunities for social engagement [7,14,15,16,17]. Studies from LMICs consistently show that caregivers of children with CP have lower social capital than caregivers of children without disabilities, particularly in rural settings where isolation and stigma are more pronounced [10,16]. These findings depict the need for evidence on appropriate interventions that stabilize the social connectedness and support systems of families with children with CP in low-resource settings like rural Bangladesh.

To overcome these barriers, a cluster-randomized controlled trial (RCT)—the “SUpporting People in extreme POverty with Rehabilitation and Therapy (SUPPORT CP)” trial was conducted in rural Bangladesh [18]. The trial evaluated whether an integrated microfinance/livelihood intervention combined with community-based rehabilitation (IMCBR) could improve children’s health-related quality of life (HRQoL) and caregivers’ socio-economic status and social capital in rural Bangladesh [18]. The primary RCT publication demonstrated improvements in HRQoL and broader social and economic outcomes among ultra-poor families receiving IMCBR [19]. However, the trial did not examine the intervention’s specific effects on the individual subdomains of social capital.

The present manuscript reports a planned secondary analysis of the SUPPORT CP trial, focusing exclusively on caregivers’ social capital. Specifically, we examine the effectiveness of the integrated intervention on social capital domains (i.e., structural and cognitive) and identify factors (e.g., socio-demographic and clinical) associated with variability in social capital outcomes among caregivers of children with CP in this rural context.

## 2. Materials and Methods

### 2.1. Study Design and Intervntion Details

Secondary analysis of data from the SUPPORT CP trial [19]. The SUPPORT CP Trial comprised of three study arms (Arm-A, Arm-B, and Arm-C). Participants in Arm-A received IMCBR; Arm-B received community-based rehabilitation (CBR) only; and Arm-C received standard care (i.e., consultation and referral to existing healthcare and rehabilitation service facilities for need-based services). A brief description of the interventions is given below.

#### 2.1.1. Arm-A: Integrated Microfinance/Livelihood Support and CBR (IMCBR)

Participants in Arm-A received livelihood support and a CBR program. The livelihood support included (i) provision of productive household assets (e.g., goat, lamb, chicken, sewing machines, and utensils for ghee production); (ii) skill development training tailored to a selected livelihood; (iii) weekly group meetings to discuss progress, challenges, and problem-solving strategies; and (iv) monthly home visits by the study team for monitoring and support. The CBR component included (i) weekly 90 min community-based sessions, comprising 60 min of goal-directed motor learning training/therapy for children with CP delivered by a trained community rehabilitation officer (CRO), (ii) 30 min of caregivers training on basic therapeutic skills for daily care aligned with goal-directed training (GDT) principles, and (iii) fortnightly home visits by CROs to deliver home-based CBR activities and assist families in creating motor-enriched play environments that promote active movement and task success.

#### 2.1.2. Arm-B: CBR Only

Participants in Arm-B received the same CBR intervention as described above (weekly 90 min sessions plus fortnightly home visits), but without the microfinance/livelihood component.

#### 2.1.3. Arm-C: Standard Care

Participants in Arm-C did not receive any active intervention. After recruitment, families were provided with basic education on early intervention and rehabilitation and were encouraged to access existing health and rehabilitation services using usual care pathways.

More details are available in our previous publication [18].

### 2.2. Study Location and Settings

The SUPPORT CP trial was conducted in three rural subdistricts (i.e., Shahjadpur, Belkuchi, and Ullahpara) of Sirajganj, located in the northern part of Bangladesh along the Jamuna River basin [20]. Sirajganj comprises a mix of peri-urban, rural, and newly formed sandbar islands known as the ‘Char’ (mid-channel island), a hard-to-reach area [20,21]. The frequent erosion and geophysical change in the land area have an unavoidable impact on the socio-economic condition of the people there [21]. The study area’s demographic characteristics, geography, with its unique blend of peri-urban, rural, and the ‘Char’ areas provides a representative setting for this research in rural Bangladesh [20].

### 2.3. Study Participants and Recruitment

Children with CP registered in the BCPR, aged 5 years or below at the time of recruitment in the trial and their primary caregivers living in the study areas were eligible to participate in the study. Twenty-four clusters were formed from mouzas, each containing 10–14 child–caregiver pairs. In Bangladesh, a mouza is the smallest administrative unit, typically comprising around five villages. These 24 clusters were randomly assigned to one of three trial arms (eight clusters per arm) using a 1:1:1 allocation ratio. To minimize contamination between intervention types, clusters were selected from three different subdistricts (i.e., Shahjadpur, Belkuchi, and Ullahpara). More details about participant recruitment are available in our study protocol [18].

### 2.4. Data Collection Method and Tools Used

The duration of the trial was 12 months. Data were collected at the baseline (month 0), midline (month 6), and endline (month 12) using a structured questionnaire developed based on existing standard tools. Information was collected based on caregiver interviews by trained assessors (research officers), direct assessment of children, and review of any available medical records. Data were collected on the following variables:

Dependent/outcome variable: The outcome variable was social capital of primary caregivers of children with CP in the study. Social capital was measured using the Shortened and Adapted Social Capital Assessment Tool (SASCAT), a widely used instrument that assesses both structural and cognitive dimensions of social capital [22,23]. We used the Bangladesh-validated version of the SASCAT, which was cognitively tested and adapted for rural and urban Bangladeshi communities (SASCAT-B) [22] (Supplementary File S1). The tool includes nine items covering (i) structural social capital—group membership, support from groups and individuals, and collective action (five items); and (ii) cognitive social capital—trust and social cohesion (four items) [22]. Each item is scored dichotomously (yes/no), with higher scores indicating the presence of the corresponding social capital component. Structural and cognitive social capital scores were derived by summing the relevant items, with higher total scores reflecting higher levels of each social capital domain [22,23].

Independent variable: Independent variables included the following:

(i) Socio-demographic and economic characteristics of children and their caregivers: Information was collected by interviewing caregivers using standard questions adapted from the BCPR case record form [12] and the Bangladesh Demographic and Health Survey (BDHS) questionnaire [24].
(ii) Motor characteristics of children: Children with CP were assessed by physiotherapists using the Gross Motor Function Classification System (GMFCS) [25] and Gross Motor Function Measure 66 items (GMFM-66) [26].
(iii) Communication function of children: Children with CP were assessed by physiotherapists using the Communication Function Classification System (CFCS) [27].
(iv) Health-related quality of life (HRQoL) of children: Caregivers were interviewed using the TNO-AZL preschool children quality of life (TAPQOL) questionnaire [28].

### 2.5. Data Analysis

The dichotomous responses (‘Yes’ and ‘No’) for individual items of both structural and cognitive social capital of the SASCAT were collected as part of the data collection process. Each response was scored as ‘1’ for ‘Yes’ and ‘0’ for ‘No’. Using this method, caregivers could score between 0 and 26 in “structural social capital items” (0–8 for group membership, 0–8 for support from groups, 0–8 for support from individuals, and 0–2 for collective action items) and between 0 and 4 in cognitive social capital items. A higher score indicated better social capital status. The total SASCAT score, as well as the combined scores for structural and cognitive social capital separately, and the scores for each of the four domains of structural social capital, were used to describe the social capital of primary caregivers [22,23].

An intention-treat analysis was performed to compare the outcomes between the three arms. Descriptive statistics were used to summarize group characteristics and assess differences between the arms. Percentage change was calculated as the ratio of absolute variation to the baseline score: (endline score − baseline score) ÷ baseline score × 100. Chi-squared tests were to assess the statistical differences in participants’ socio-demographic and clinical characteristics between the trial arms. Additionally, an analysis of covariance (ANCOVA) test with Bonferroni adjustment was used to test the differences in SASCAT scores between the study arms. Clustering was accounted for by fitting mixed-effects models in which the intervention arm was included as a fixed effect and participants were nested within clusters as a random effect. A multivariable linear regression model was developed to identify the predictors of SASCAT scores at the endline, adjusting for baseline SASCAT data. All analyses were conducted in SPSS (version 26), with statistical significance set at *p* < 0.05.

### 2.6. Ethical Consideration

The trial was registered with the Australian New Zealand Clinical Trials Registry (ACTRN12619001750178). Ethics approval was obtained from the Human Research Ethics Committee of the Asian Institute of Disability and Development (reference number: SOUTHASIA-HREC-2019-5-03) and the Bangladesh Medical Research Council (BMRC) (reference number: BMRC/NREC/2016–2019/251; registration number: 224 1706 2019). Informed written consents were obtained from each caregiver prior to recruitment. A participant information sheet (written in Bangla, the local language) was provided to each participant.

## 3. Results

**Figure 1:**
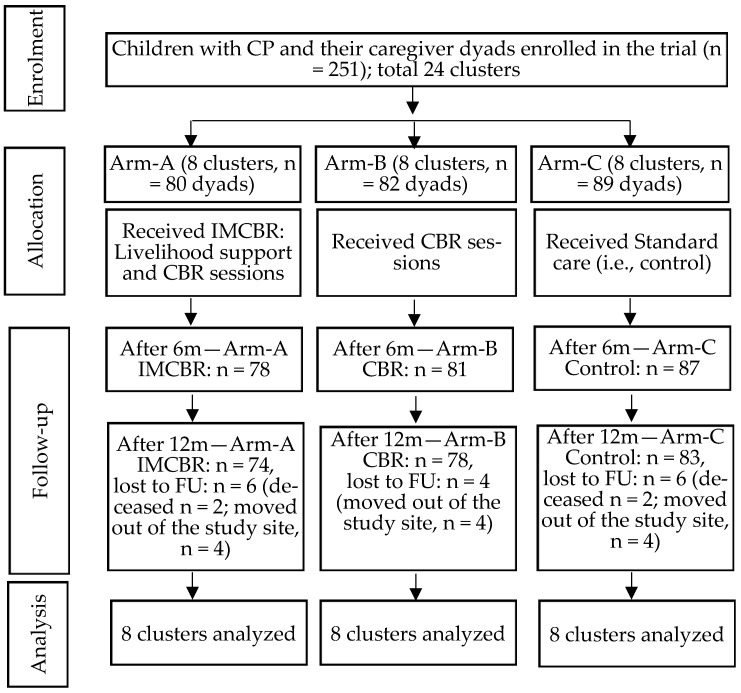
Study diagram.

A total of 251 children with CP and their primary caregivers were enrolled in the trial and randomly assigned to one of three arms: Arm-A (*n* = 80), Arm-B (*n* = 82), and Arm-C (*n* = 89), with each arm comprising eight clusters. The majority of primary caregivers were mothers (95.6%). Of the enrolled children, 235 children completed the endline assessments (92.8%) (Figure 1).

### 3.1. Socio-Demographic Characteristics

The mean (SD) age of the mothers was 24.2 (5.6) years. Most of the mothers completed a primary education level, and this was consistent across Arm-A, Arm-B, and Arm-C (*p* = 0.499). A similar pattern was observed for the education level of the fathers of the children with CP (*p* = 0.224). The majority of the mothers were not involved in any income-generating activities (IGA), with no significant differences across study Arm-A, Arm-B, and Arm-C (*p* = 0.449), whereas the contrary was observed for their husbands/father of children with CP. Most of the fathers of children with CP in Arm-A and Arm-B were involved in different professional/skilled/unskilled jobs (67.5 and 64.6%, respectively), whereas in Arm-C, the majority were involved in agricultural/farming (41.6%) (*p* < 0.001) (Table 1).

There were significant baseline differences in monthly household income and socio-economic status (SES) among the study arms. Low SES and low monthly income were more common in families in Arm-A compared to those in Arm-B and Arm-C (*p* < 0.05 for both) (Table 1).

No significant difference was observed in gender and the motor characteristics of children with CP across the study’s arms (*p* > 0.05 for both); however, children in Arm-B and Arm-C had a slightly higher proportion of severe communication difficulties compared to children in Arm-A (CFCS level III–V: 84.1% and 87.6%, respectively, vs. 72.5%, *p* = 0.031).

### 3.2. Social Capital Score

At baseline, the total social capital score was slightly lower among caregivers in Arm-A compared to Arm-B and Arm-C. This was consistent for all domains of structural and cognitive social capital.

When compared between the baseline and endline, the percentage increase in total score was the highest in Arm-A (60.0%), followed by Arm-B (54.1%) and Arm-C (6.0%). The between-group differences were statistically significant for Arm-A vs. Arm-C and Arm-B vs. Arm-C (both *p* < 0.001), but not between Arm-A and Arm-B (*p* = 0.395). (Table 2).

A similar trend was observed for structural social capital. Caregivers in Arm-A had the greatest percentage increase (135.7%), followed by Arm-B (96.9%) and Arm-C (8.1%). Between-group differences were significant for Arm-A vs. Arm-C and Arm-B vs. Arm-C (both *p* < 0.001). For cognitive social capital, the highest percentage increase was seen in Arm-B (6.9%), followed by Arm-C (3.3%) and Arm-A (−6.3%). However, differences in score changes across the three arms were not statistically significant (*p* > 0.05 for all) (Table 2). Sensitivity analyses adjusting for baseline socio-economic variables produced estimates that were consistent with the primary model, with between-group differences remaining stable across all covariate-adjusted analyses (Supplementary File S2).

### 3.3. Factors Related to Changes in Social Capital Score

After adjusting for baseline values, endline social capital scores were negatively associated with caregiver–child relationship, mother’s age, child’s gender, and the child’s CFCS level in all three arms. However, none of these associations were statistically significant (*p* > 0.05 for all). The GMFCS level of the child showed a significant negative association with total social capital score in Arm-A (*p* = 0.023); however, it showed a non-significant negative association in Arm-B (*p* = 0.546) and Arm-C (*p* = 0.814) (Table 3).

## 4. Discussion

This study demonstrated that both IMCBR and CBR alone substantially improved caregivers’ social capital relative to standard care. IMCBR showed the largest gains in overall and structural social capital scores, although differences between IMCBR and CBR only were directional rather than statistically significant for the total score and most subdomains. These findings suggest that CBR is effective at strengthening the social networks and participation of caregivers, while the addition of livelihood support may further enhance these benefits, even if not always at statistically detectable levels within the study’s timeframe.

In our study, improvements in cognitive social capital were modest across all arms. This indicates that cognitive dimensions reflecting norms, shared values, and perceptions of trust typically change differently than tangible, behavior-based forms of social capital. The possible explanations include high baseline levels (ceiling effects) in these rural settings, the need for longer and more stable community engagement to shift attitudes, and the fact that cognitive social capital is often shaped by broader social norms that may not respond quickly to short-term interventions [29,30,31].

The inverse relationship between child’s motor severity (GMFCS level) and caregiver’s social capital in the IMCBR arm is particularly noteworthy. Previous studies have consistently demonstrated that the caregivers of children with severe motor impairments experience heightened caregiving burden, stress, and perceive lower levels of social support [8,9,32]. The caregivers may also face greater time and energy constraints, limiting their ability to attend group meetings, participate in livelihood activities, or engage in community life. Furthermore, stigma related to visible or severe disability can further reduce caregivers’ social interactions and perceived acceptance within communities [8]. These interacting factors may collectively erode their social capital. Notably, in our study, this association was not observed in Arm-C, highlighting potential contextual differences in how the severity of disability intersects with caregiver networks and resources. These findings reinforce the need for tailored adaptations in livelihood and CBR programs such as respite care, assistive devices, flexible group formats, transport support, and home-based options to ensure equitable social participation for families of children with severe motor impairments.

The lack of significant associations between social capital and socio-demographic factors, such as parental education and SES contrasts with the findings from a few previous studies [3,32]. One possible explanation is the relatively homogenous socio-economic profile of this study’s population, which minimized the variability needed to detect significant effects. It is also plausible that in this rural Bangladeshi context, community dynamics and shared cultural norms play a more dominant role in shaping social capital, overshadowing the influence of individual socio-demographic characteristics.

The significant improvements in social capital observed in IMCBR and CBR groups, as opposed to the ‘standard care’ group, suggest that targeted interventions can effectively enhance social capital among caregivers. Structural social capital, group membership, and social support showed particularly strong improvements, indicating the potential benefits of community-based programs that foster peer support and group participation. The findings highlight the importance of designing interventions that provide direct support and facilitate the creation of supportive social networks for caregivers.

A key limitation of this trial was the disruption caused by the COVID-19 pandemic, which led to an incomplete implementation of the intervention. As a result, the program could not proceed as per protocol [19], and significant adjustments were necessary midway through the study. Additionally, the baseline varied household income across the study arms, particularly families in Arm-A, which received the integrated intervention, were the most economically disadvantaged. Employing stratified random sampling in future studies—particularly by distinguishing between urban and rural clusters—may help to address such baseline imbalances. It is also important to acknowledge that not all livelihood initiatives yield immediate financial returns, especially within a relatively short period such as 12 months. Despite these challenges, the trial offered valuable insights into how livelihood support can be meaningfully combined with conventional CBR efforts and improve the social capital of caregivers of children with CP in rural LMICs like Bangladesh. Lessons from this trial can inform the design of future large-scale studies and implementation research in similar contexts.

## 5. Conclusions

This study demonstrates that CBR, along with livelihood support, can substantially enhance social capital among the caregivers of children with CP in rural settings. Improvements were most evident in structural social capital, while gains in cognitive social capital were smaller. The negative association between social capital gains and severe motor impairment underscores the need for tailored strategies to support caregivers of children with severe motor impairments. Strengthening social support networks and fostering inclusive community engagement may help ensure more equitable social capital benefits for families and caregivers.

## Figures and Tables

**Table 1:** Characteristics of mother/primary caregiver of participating children with disabilities.

Factors	Total	Study Arm			*p* Value
		Arm-A	Arm-B	Arm-C	
Caregiver’s relationship with the child					
Mother	240 (95.6)	77 (96.3)	78 (95.1)	85 (95.5)	1.000 ^3^
Others	11 (4.4)	3 (3.8)	4 (4.9)	4 (4.5)	
Mother’s age in years					
20 years or less	84 (33.6)	20 (25.0)	35 (42.7)	29 (33.0)	0.012 ^1^
21–30 years	125 (50.0)	52 (65.0)	31 (37.8)	42 (47.7)	
31–40 years	41 (16.4)	8 (10.0)	16 (19.5)	17 (19.3)	
Mean (SD)	24.2 (5.6)	24.30 (5.3)	24.00 (6.4)	24.34 (5.8)	0.920 ^2^
Mother’s educational level					
No formal schooling/incomplete primary level	66 (26.3)	24 (30.0)	16 (19.5)	26 (29.2)	0.499 ^1^
Completed primary	154 (61.4)	48 (60.0)	55 (67.1)	51 (57.3)	
Completed secondary and higher	31 (12.4)	8 (10.0)	11 (13.4)	12 (13.5)	
Father’s educational level					
No formal schooling/incomplete primary level	85 (33.9)	35 (43.8)	24 (29.3)	26 (29.2)	0.224 ^1^
Completed primary	131 (52.2)	37 (46.3)	46 (56.1)	48 (53.9)	
Completed secondary and higher	35 (13.9)	8 (10.0)	12 (14.6)	15 (16.9)	
Mother’s occupation					
Job (professional/skilled/unskilled)	13 (5.2)	6 (7.5)	3 (3.7)	4 (4.5)	0.449 ^3^
Business	1 (0.4)	0 (0.0)	1 (1.2)	0 (0.0)	
Agriculture/farming	3 (1.2)	1 (1.3)	2 (2.4)	0 (0.0)	
Housewife	234 (93.2)	73 (91.3)	76 (92.7)	85 (95.5)	
Father’s occupation					
Job (professional/skilled/unskilled)	143 (57.0)	54 (67.5)	53 (64.6)	36 (40.4)	<0.001 ^1^
Business	43 (17.1)	10 (12.5)	17 (20.7)	16 (18.0)	
Agriculture/farming	65 (25.9)	16 (20.0)	12 (14.6)	37 (41.6)	
Socio-economic status (SES) level					
Low SES	100 (40.0)	45 (56.3)	25 (30.5)	30 (34.1)	0.005 ^1^
Middle SES	100 (40.0)	22 (27.5)	36 (43.9)	42 (47.7)	
High SES	50 (20.0)	13 (16.3)	21 (25.6)	16 (18.2)	
Monthly family income (in BDT ~ USD)					
5000–7275 (63–91)	62 (25.0)	30 (39.0)	14 (17.1)	18 (20.2)	0.004 ^1^
7276–10,000 (91–125)	80 (32.3)	26 (33.8)	31 (37.8)	23 (25.8)	
10,001–15,000 (125–188)	57 (23.0)	12 (15.6)	17 (20.7)	28 (31.5)	
15,001 to 100,000 (188–1250)	49 (19.8)	9 (11.7)	20 (24.4)	20 (22.5)	
Mean (SD)	12,509 (9309) ~156 (116)	10,274 (7036) ~134 (88)	14,079 (12,187) ~176 (152)	12,994 (7543) ~162 (94)	0.029 ^2^
Child’s age in years					
<2 y	33 (13.3)	15 (19.0)	8 (9.8)	10 (11.4)	0.486 ^3^
2–4 y	207 (83.1)	61 (77.2)	71 (86.6)	75 (85.2)	
5 and above	9 (3.6)	3 (3.8)	3 (3.7)	3 (3.4)	
Mean (SD)	3.4 (1.1)	3.4 (1.2)	3.3 (1.0)	3.4 (1.1)	0.859 ^2^
Child’s gender					
Male	149 (59.4)	47 (58.8)	43 (52.4)	59 (66.3)	0.181 ^1^
Female	102 (40.6)	33 (41.3)	39 (47.6)	30 (33.7)	
Child’s GMFCS level at baseline					
GMFCS I–II	62 (24.7)	25 (31.3)	18 (22.0)	19 (21.3)	0.257 ^1^
GMFCS III–V	189 (75.3)	55 (68.8)	64 (78.0)	70 (78.7)	
Child’s CFCS level at baseline					
CFCS I–II	46 (18.3)	22 (27.5)	13 (15.9)	11 (12.4)	0.031 ^1^
CFCS III–V	205 (81.7)	58 (72.5)	69 (84.1)	78 (87.6)	

^1^ Chi-squared test; ^2^ ANOVA; ^3^ Fisher’s Exact test; *p* < 0.05 denotes statistical significance.

**Table 2:** Social capital score of mothers/primary caregivers of participating children with disability.

Social Capital Score (Mean, SD)	Baseline	Midline	Endline	Within-Group Change (% Change Between Baseline and Endline) ^1^	Between-Group Change, Mean Difference (95% CI), *p* ^2^
Total score					
Arm-A	6.0 (1.5)	10.1 (1.3)	9.6 (1.2)	60.0%	Arm-A–B: 0.3 [−0.2, 0.7], 0.395
Arm-B	6.1 (1.4)	10.3 (1.5)	9.4 (1.2)	54.1%	Arm-B–C: 2.5 [2.1, 2.9], <0.001
Arm-C	6.7 (1.3)	6.7 (1.2)	7.1 (1.2)	6.0%	Arm-A–C: 2.8 [2.3, 3.2], <0.001
Structural social capital					
Arm-A	2.8 (1.3)	7.0 (1.2)	6.6 (1.2)	135.7%	Arm-A–B: 0.4 [−0.0, 0.8], 0.052
Arm-B	3.2 (1.3)	7.0 (1.2)	6.3 (1.1)	96.9%	Arm-B–C: 2.5 [2.0, 2.9], <0.001
Arm-C	3.7 (1.3)	3.6 (1.1)	4.0 (1.2)	8.1%	Arm-A–C: 2.9 [2.4, 3.3], <0.001
Group membership					
Arm-A	0.5 (0.7)	1.6 (0.5)	1.5 (0.6)	200.0%	Arm-A–B: 0.1 [−0.1, 0.3], 0.536
Arm-B	0.6 (0.5)	1.6 (0.5)	1.5 (0.6)	150.0%	Arm-B–C: 0.8 [0.6, 0.9], <0.001
Arm-C	0.6 (0.5)	0.7 (0.5)	0.7 (0.5)	16.7%	Arm-A–C: 0.9 [0.7, 1.0], <0.001
Social support					
Arm-A	1.8 (0.8)	5.1 (0.7)	4.6 (0.5)	155.6%	Arm-A–B: 0.2 [−0.0, 0.4], 0.185
Arm-B	2.3 (0.7)	5.0 (0.7)	4.5 (0.6)	95.7%	Arm-B–C: 1.8 [1.6, 2.0], <0.001
Arm-C	2.6 (0.8)	2.7 (0.5)	2.8 (0.5)	7.7%	Arm-A–C: 2.0 [1.8, 2.2], <0.001
Collective action					
Arm-A	0.5 (0.6)	0.4 (0.5)	0.4 (0.5)	−20.0%	Arm-A–B: 0.2 [−0.0, 0.4], 0.141
Arm-B	0.3 (0.5)	0.4 (0.6)	0.3 (0.5)	0.0%	Arm-B–C: −0.3 [−0.5, 0.1], 0.007
Arm-C	0.4 (0.6)	0.3 (0.4)	0.5 (0.6)	25.0%	Arm-A–C: −0.1 [−0.3, 0.1], 0.933
Cognitive social capital					
Arm-A	3.2 (0.7)	3.1 (0.2)	3.0 (0.2)	−6.3%	Arm-A–B: −0.1 [−0.2, 0.0], 0.582
Arm-B	2.9 (0.5)	3.3 (0.4)	3.1 (0.3)	6.9%	Arm-B–C: 0.0 [−0.1, 0.1], 1.000
Arm-C	3.0 (0.4)	3.1 (0.3)	3.1 (0.3)	3.3%	Arm-A–C: −0.0 [−0.1, 0.1], 0.744
Trust					
Arm-A	1.2 (0.6)	1.1 (0.2)	1.0 (0.2)	−16.7%	Arm-A–B: −0.1 [−0.2, 0.0], 0.507
Arm-B	0.9 (0.5)	1.3 (0.4)	1.1 (0.3)	22.2%	Arm-B–C: −0.0 [−0.1, 0.1], 1.000
Arm-C	1.0 (0.4)	1.1 (0.3)	1.1 (0.3)	10.0%	Arm-A–C: −0.1 [−0.2, 0.0], 0.431
Social cohesion					
Arm-A	2.0 (0.1)	2.0 (0.0)	2.0 (0.0)	0.0%	Arm-A–B: −0.0 [−0.0, 0.0], 1.000
Arm-B	2 (0.0)	2.0 (0.0)	2.0 (0.0)	0.0%	Arm-B–C: 0.0 [−0.0, 0.0], 0.731
Arm-C	2 (0.0)	2.0 (0.0)	2.0 (0.1)	0.0%	Arm-A–C: 0.0 [−0.0, 0.0], 0.755

^1^ Percentage change was calculated as the ratio of absolute variation to the baseline score: (endline score − baseline score) ÷ baseline score × 100. ^2^ Analysis of covariance (ANCOVA); *p* < 0.05 denotes statistical significance.

**Table 3:** Factors related to social capital score of caregivers of children with CP in the study.

Covariates	Study Arm					
	Arm-A		Arm-B		Arm-C	
	*β* (95% CI) ^1^	*p*-Value	β (95% CI) ^1^	*p*-Value	β (95% CI) ^1^	*p*-Value
Caregiver’s relationship with the child: Mother	1.0 [−0.4, 2.4]	0.144	0.5 [−0.5, 1.6]	0.333	0.5 [−0.7, 1.7]	0.379
Caregiver’s age: 21 years and over	−0.3 [−1.0, 0.3]	0.291	0.1 [−0.4, 0.6]	0.759	−0.5 [−1.0, 0.0]	0.058
Mother’s educational level: Literate	0.1 [−0.5, 0.8]	0.638	0.3 [−0.3, 0.9]	0.282	−0.4 [−0.9, 0.2]	0.194
Father’s educational level: Literate	−0.4 [−1.0, 0.1]	0.136	0.1 [−0.4, 0.6]	0.662	0.2 [−0.3, 0.8]	0.442
Mother’s occupation: Housewife	0.0 [−0.9, 1.0]	0.932	0.1 [−0.7, 1.0]	0.737	−0.6 [−1.8, 0.5]	0.286
Father’s occupation: Self employed	−0.2 [−0.8, 0.4]	0.523	−0.2 [−0.7, 0.3]	0.366	−0.2 [−0.3, 0.7]	0.377
Socio-economic status (SES): Mid-High SES	−0.2 [−0.4, 0.8]	0.515	0.3 [−0.2, 0.8]	0.169	−0.2 [−0.7, 0.3]	0.436
Monthly family income: > 10,000 BDT (> 125 USD)	0.0 [−0.6, 0.6]	0.946	0.1 [−0.3, 0.6]	0.568	0.5 [−0.0, 1.0]	0.051
Child’s age: 3 years and above	0.5 [−0.0, 1.1]	0.052	0.2 [−0.3, 0.7]	0.384	0.4 [−0.2, 0.9]	0.168
Child’s gender: Female	−0.2 [−0.8, 0.4]	0.456	−0.0 [−0.5, 0.4]	0.887	−0.2 [−0.7, 0.4]	0.514
Child’s GMFCS level at baseline: III-V	−0.7 [−1.3, −0.1]	0.023	−0.2 [−0.7, 0.4]	0.546	0.1 [−0.5, 0.7]	0.814
Child’s CFCS level at baseline: III-V	−0.4 [−1.1, 0.2]	0.175	−0.2 [−0.8, 0.5]	0.673	−0.1 [−0.9, 0.6]	0.770

^1^ Adjusted for total SASCAT score at baseline; *p* < 0.05 denotes statistical significance.

## Data Availability

The data are not publicly available for ethical reasons but can be accessed upon request from the corresponding author.

## Supplementary Materials

The following supporting information can be downloaded at: https://www.mdpi.com/article/10.3390/children12121687/s1, File S1: Shortened and Adapted Social Capital Assessment Tool–Bangladesh; File S2: Sensitivity analysis.
