# Supplementary material for: Effectiveness of an Integrated Community-Based Livelihood and Rehabilitation Intervention on the Social Capital of Caregivers of Children with Cerebral Palsy: Secondary Analysis of an Existing Cluster Randomized Controlled Trial in Rural Bangladesh
Source: Children (Basel). 2025 Dec 11;12(12):1687. doi: 10.3390/children12121687 (PMC12731373; doi:10.3390/children12121687)
Supplement: Supplementary file 1 [file children-12-01687-s001.zip › children-3976185-supplementary.pdf]

Supplementary File S1:

Shortened and Adapted Social Capital Assessment Tool - Bangladesh

Structural Social Capital

Group membership

1a. In the last 12 months, have you been a member of the following types of groups in your area?

- Vocational training group
- Savings group/community cooperative
- Political group
- Religious group
- Microcredit program
- Sports club
- Youth/student club
- Other: specify

1b. In the last 12 months, how would you describe your involvement in the groups in which you are a member?

- Received a loan or other form of financial support
- Attended meetings
- Attended trainings
- Participated in decision making
- Served as a leader of the group
- Other: specify

Social support

2a. Suppose you had something unfortunate happen to you, such as a father's sudden death. Who would help you in this situation?

- Immediate family
- Relatives
- Neighbors
- Friends who are not neighbors
- Community leaders
- Religious leaders
- Politicians
- Government officials/civil service
- Person from NGO
- A group in which I am a member
- A group in which I am not a member
- Other: specify

2b. Suppose you suffered an economic loss, such as job loss (urban)/crop failure (rural). In that situation, who do you think would assist you financially[a]?

2c. Suppose you are (female)/your wife is (male) preparing to give birth to your (female)/her (male) first child. Who do you think would provide you (female)/her (male) advice or assistance in this situation[a]?

Collective action

3. In the last 12 months, have you joined together with others in your area to address important issues?
   - Yes
   - No
4. In the last 12 months, have you talked with a local leader, chairman, or governmental organization about the development of your area?
   - Yes
   - No

Cognitive Social Capital

Trust

5a. Can your neighbors be trusted?

- Yes
- Sometimes
- No

5b. Can leaders in this area be trusted?

- Yes
- Sometimes
- No

6. Do you think that the majority of people in this area would try to take advantage of you if they got the chance?
   - Yes
   - Sometimes
   - No

Social cohesion

7. Do the majority of people in this area generally have good relationships with each other?
   - Yes
   - Sometimes
   - No
8. Do you feel that this area is yours?
   - Yes
   - Sometimes
   - No

Note. NGO = nongovernmental organizations.

[a]Use the same list of response options as in Question 2a.

Supplementary File S2:

Sensitivity analysis

**ANCOVA test with mother's age as a covariate**	
Study Arm	Between group change (mean difference, 95% CI, p value)
Arm A-B	0.29 [-0.16, 0.73], 0.356
Arm B-C	2.48 [2.04, 2.92], **<0.001**
Arm A-C	2.77 [2.32, 3.21], **<0.001**
**ANCOVA test with monthly family income as a covariate**	
Study Arm	Between group change (mean difference, 95% CI, p value)
Arm A-B	0.25 [-0.19, 0.70], 0.523
Arm B-C	2.52 [2.07, 2.96], **<0.001**
Arm A-C	2.77 [2.32, 3.22], **<0.001**
**ANCOVA test with father's occupation as a covariate**	
Study Arm	Between group change (mean difference, 95% CI, p value)
Arm A-B	0.29 [-0.17, 0.72], 0.395
Arm B-C	2.48 [2.03, 2.94], **<0.001**
Arm A-C	2.76 [2.30, 3.22], **<0.001**
**ANCOVA test with socioeconomic status as a covariate**	
Study Arm	Between group change (mean difference, 95% CI, p value)
Arm A-B	0.26 [-0.19, 0.71], 0.496
Arm B-C	2.50 [2.06, 2.94], **<0.001**
Arm A-C	2.76 [2.31, 3.21], **<0.001**
**ANCOVA test with CFCS level as a covariate**	
Study Arm	Between group change (mean difference, 95% CI, p value)
Arm A-B	0.26 [-0.19, 0.70], 0.507
Arm B-C	2.48 [2.05, 2.92], **<0.001**
Arm A-C	2.74 [2.29, 3.19], **<0.001**
**ANCOVA test with mothers' age, father's occupation, monthly family income, socioeconomic status and CFCS level as covariates**	
Study Arm	Between group change (mean difference, 95% CI, p value)
Arm A-B	0.31 [-0.15, 0.78], 0.314
Arm B-C	2.50 [2.04, 2.96], **<0.001**
Arm A-C	2.81 [2.33, 3.30], **<0.001**
